# Fetal growth standards for Chinese twin pregnancies

**DOI:** 10.1186/s12884-021-03926-y

**Published:** 2021-06-22

**Authors:** Jianping Chen, Jun Zhang, Yang Liu, Xing Wei, Yingjun Yang, Gang Zou, Yun Zhang, Tao Duan, Luming Sun

**Affiliations:** 1 Department of Fetal Medicine and Prenatal Diagnosis Center, Shanghai First Maternity and Infant Hospital, Tongji University School of Medicine, 2699 West Gaoke Rd, Shanghai, 201204 China; 2 Ministry of Education-Shanghai Key Laboratory of Children’s Environmental Health, Xinhua Hospital, Shanghai Jiao Tong University School of Medicine, Shanghai, China

**Keywords:** Fetal growth standards, Twin pregnancies, Ultrasound-based, Chinese

## Abstract

**Background:**

The common use of singleton fetal growth standard to access twin growth might lead to over-monitoring and treatment. We aimed to develop fetal growth standards for Chinese twins based on ultrasound measurements, and compare it with Zhang’s and other twin fetal growth charts.

**Methods:**

A cohort of uncomplicated twin pregnancies were prospectively followed in 2014–2017. Smoothed estimates of fetal growth percentiles for both monochorionic (MC) and dichorionic (DC) twins were obtained using a linear mixed model. We also created growth charts for twins using a model-based approach proposed by Zhang et al. Our twin standards were compared with Hadlock’s (singleton) in predicting adverse perinatal outcomes.

**Results:**

A total of 398 twin pregnancies were included, with 214 MC and 582 DC live-born twins. The MC twins were slightly lighter than the DC twins, with small differences throughout the gestation. Our ultrasound-based fetal weight standards were comparable to that using Zhang’s method. Compared with previous references/standards from the US, Brazil, Italy and UK, our twins had very similar 50th percentiles, but narrower ranges between the 5th and 95th or 10th and 90th percentiles. Compared with the Hadlock’s standard, the risks of neonatal death and adverse perinatal outcomes for small for gestational age (SGA) versus non-SGA were substantially elevated using our standards.

**Conclusions:**

A normal fetal growth standard for Chinese twins was created. The differences between MC and DC twins were clinically insignificant. The 50th weight percentiles of the Chinese twins were identical to those in other races/ethnicities but the ranges were markedly narrower. Our standard performed much better than the Hadlock’s in predicting low birth weight infants associated with adverse perinatal outcomes in twin pregnancies. The present study also indicated that Zhang’s method is applicable to Chinese twins, and other areas may use Zhang’s method to generate their own curves for twins if deemed necessary.

**Supplementary Information:**

The online version contains supplementary material available at 10.1186/s12884-021-03926-y.

## Background

Thanks to the development of assisted reproductive technology and delayed childbearing, the incidence of twin pregnancies rose steadily in the last four decades. The twinning rate is now estimated at around 2 ~ 3% in all pregnancies [1, 2]. Twin pregnancies are at higher risks of multiple adverse perinatal outcomes than singleton pregnancies, mainly due to prematurity and/or fetal growth restriction (FGR) [3, 4]. Thus, identifying fetuses with growth restriction is crucial in prenatal care of twin pregnancies.

It is a common clinical practice to evaluate twin growth status using a fetal growth chart that was developed for singleton pregnancies. Twin and singleton fetuses may follow similar growth patterns during the first and second trimesters [5], but the growth of twins slows down in the third trimester, and the growth curves between twin and singleton pregnancies diverge significantly after 28–32 weeks gestation and the difference between them widens with advancing gestation [6–8]. Whether the growth difference between singletons and twins is a pathological consequence(real growth problem) or a physiological adaption remains controversial. However, it has now been well-recognized that the growth of twins lags behind that of singletons especially at late gestation. Therefore, using singleton standards for twins may identify more SGA fetuses especially at late gestation, leading to over-monitoring and treatment, and increasing medical burden and costs. It is now widely acknowledged that singletons and twins need separate growth charts to assess their growth accurately [9].

Some studies have tried to establish fetal growth charts for twins from population-based birthweight [7, 10–12]. However, as infants born prematurely are more likely to be growth restricted than fetuses who remain in utero at the same gestational age [13], a birthweight-based chart would underestimate the proportion of FGR before term. In the past decade, several fetal growth charts for twins based on ultrasonography measurements have been created, some of which were stratified by chorionity [8, 14–17].

At the same time, Zhang et al. proposed a method to develop an adjustable fetal weight standard for twins [18]. It adopts the Gardosi’s proportionally principle [19], and assumes that the standard deviation is a constant fraction of the mean weight through gestation [20]. Based on the theory, by anchoring to the mean birth weight and standard deviation of a specific gestation age (i.e. 37.5 weeks), corresponding percentiles across each gestational age can be calculated based on normal distribution following Hadlock’s formula [21]. To date, there was no ultrasound-based growth chart specially built for Chinese twins. Also, the effectiveness of Zhang’s method needs to be validated.

Our study aimed to construct a fetal growth chart for Chinese twins based on ultrasound biometric measurements, and compare it with Zhang’s and other twin fetal growth charts for validation [8, 14–17].

## Methods

### Population

This study used data from a prospective study on preeclampsia screening in twin pregnancies. A total of 1475 women were approached and 1225 were enrolled between gestation of 11 weeks 0 days and 13 weeks 6 days and followed to delivery or end of pregnancy at the Shanghai Frist Maternity and Infant Hospital in 2014–2017 [22]. At enrollment, an ultrasound scan was conducted for each twin. Ultrasound-estimated gestational age (Us-GA) was calculated based on the fetal crown-rump length of the larger twin using the formula by Robinson and Fleming: Us-GA (in exact weeks) = (8.052*$\sqrt{\mathrm{C}\mathrm{R}\mathrm{L}}$+23.73)/7 [23], and chorionicity was determined by the presence of T sign (monochorionic diamniotic, MCDA) or λ sign (dichorionic diamniotic, DCDA) at the junction site of intertwin membrane with the placenta. Pregnancies with uncertain chorionicity were not eligible for preeclampsia screening study. Written informed consents were obtained from all participants.

In the present study, we firstly excluded pregnancies with unmatched Us-GA and last menstrual period-based gestational age (LMP-GA) (*n* = 32), in which the difference between Us-GA and LMP-GA were: 1) more than 6 days for gestation estimates between 11 weeks 0 days and 12 weeks 6 days of gestation; or 2) more than 7 days between 13 weeks 0 days and 13 weeks 6 days. For those conceived by in vitro fertilization (IVF), the last menstrual period (LMP) was calculated by the date of transfer minus 14 days and embryo age at transfer. LMP-GA was used as the gestational age in all analyses.

**Fig. 1:**
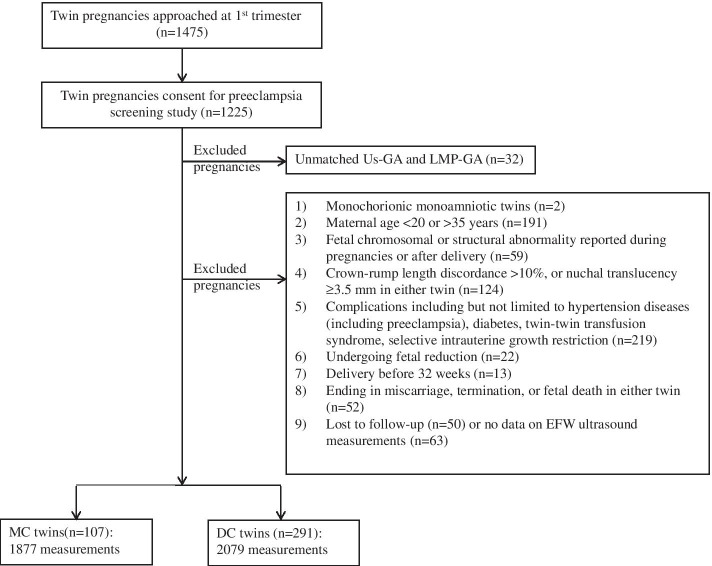
Flow chart for the study population

We further excluded pregnancies: 1) with monochorionic monoamniotic twins (*n* = 2); 2) with maternal age < 20 or > 35 years (*n* = 191); 3) with fetal chromosomal or major structural abnormality reported during pregnancies or after delivery (*n* = 59); 4) with crown-rump length discordance > 10%, or nuchal translucency ≥ 3.5 mm in either twin (*n* = 124); 5) with complications including but not limited to hypertensive disorders (including preeclampsia), diabetes, twin-twin transfusion syndrome(TTTS), selective intrauterine growth restriction (sIUGR, defined as estimated fetal weight < 10th percentile in the small fetus and weight discordance ≥ 25% between the two fetuses) (*n* = 219); 5) undergoing fetal reduction (*n* = 22); 6) delivery before 32 weeks (*n* = 13); 7) ending in miscarriage, termination, or fetal death in either twin (*n* = 52); or 8) being lost to follow-up (*n* = 50) or having no data on ultrasound biometric measurements (*n* = 63). In this way, we aimed to select only healthy women who were at a better condition for optimal fetal growth and only healthy fetuses who were considered to have an optimal growth, and to construct an optimal growth standard for twin-fetuses. The flow chart for the study population was presented in Fig. 1.

### Maternal characteristics and birth weight

Maternal characteristics and medical history were recorded, including maternal age, weight, height, parity (nulliparous or parous), method of conception (spontaneous conception, ovulation induction, and in vitro fertilization). The birth weight of the twins was measured by electronic baby balance and recorded immediately after birth.

### Ultrasound biometric measurements

Transabdominal ultrasound scans of fetal biometric measurements were conducted by 3 certified sonographers in the Department of Fetal Medicine at the Shanghai First Maternity and Infant Hospital, who were specially trained and had experience in obstetrical and fetal ultrasonography. All scans were performed on the Voluson E8 machines (GE Healthcare Ultrasound Milwaukee, WI, USA). At the first scan, twin A or twin B was accurately labeled using the placental site, fetal position (up or down; right or left), and cord insertion. For each fetus, biparietal diameter (BPD), head circumference (HC), abdominal circumference (AC), and femur length (FL) were measured according to the ISUOG Guideline [24]. Each biometric index was measured twice, and the average was calculated. Estimated fetal weight (EFW) was calculated using ultrasound biometric parameters by Hadlock formula IV: Log_10_ weight = 1.3596–0.00386 × AC × FL + 0.0064 × HC + 0.00061 × BPD × AC + 0.0424 × AC + 0.174 × FL [25]. Measurements were excluded if the EFW was unreasonable, defined as greater than 5 standard deviations from the mean.

### Statistical analysis

**Fig. 2:**
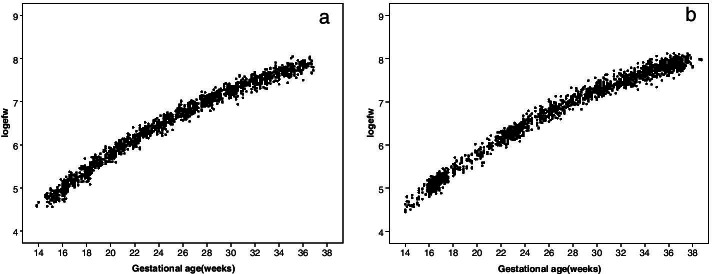
Scatter plot of log-transformed estimated fetal weight vs. gestational age in MC (**a**) and DC twins (**b**)

Smoothed estimates of fetal growth chart and percentiles for both monochorionic (MC) and dichorionic (DC) twins were obtained using linear mixed models, which could account for the dependency of the data, including clustering of the twins and serial measurements on the same fetus. The EFW measurements were log-transformed to ensure the homoscedasticity of variance across gestational age and the normal distribution of EFW at each gestational age. We included random effects for both mother (twin-pair) and individual fetus (serial EFW measurements, fetus-level). For the modeling procedure, we tested for models of log-transformed EFW on gestational age, gestational age squared and gestational age cubed. The best fit model was selected based on the lowest Akaike information criteria (AIC) value and residual standard errors (Supplementary Table S1). Finally, the model of log-transformed EFW on gestational age, gestational age squared was selected. The scatters of log-transformed EFW against gestational age were plotted in Fig. 2 (a, MC twins; b, DC twins).

Gestational age specific percentiles for fetal weight were calculated on the log scale and then back-transformed to the original fetal weight scale in grams. The gestational age specific variance was estimated by combining the estimated twin-pair level, fetus-level and residual variance, and the corresponding standard deviation (SD) was then estimated by regressing the squared root of the gestational age specific variance on gestational age [17]. We assumed a normal distribution of the log fetal weight on each gestational age, and used the formula *Mean* ± *Z*α × *SD* to obtain the log scale percentile, where *Mean* is the predicted value of the optimal model, *Zα* is the corresponding value for the percentile of the standard Gaussian distribution, and *SD* is gestational age specific standard deviation [20]. Based on the same method, the standards for twin fetal biometric measurements (BPD, HC, AC, and FL) were also calculated. However, with the model of log-transformed measurement on gestational age, gestational age squared, and gestational age cubed being selected.

In order to investigate whether the fetal growth is different between pregnancies conceived naturally and pregnancies conceived by in vitro fertilization, we added a sensitivity analysis and compared the fetal growth charts between the two sub-populations. At the same time, for comparison, following Zhang’s method [18], we created a growth chart for twins by anchoring to the mean birth weight and standard deviation at the gestational age of 37 weeks in the study population (37 + 0 to 37 + 6 weeks, 356 fetuses, 2709.8 ± 274.0 g).

To assess the performance of the growth chart in identifying the true “small” fetuses who were at higher risk of adverse perinatal outcomes, we applied the established chart to live-born twins of the source cohort, and compared it with Hadlock’s singleton standard. Among the 1225 twin pregnancies enrolled for preeclampsia screening, 1091 women delivered 2 live births, of which 1920 births had complete perinatal information. The odds ratios (ORs) of neonatal death and adverse perinatal outcomes between small for gestational (SGA) and non-SGA infants were estimated. The neonatal death was defined as death within 7 days after birth, and adverse perinatal outcomes included neonatal death, neonatal intensive care unit (NICU) stay for ≥ 14d, and transfer to a higher-level or special care unit.

### Role of funding source

The funders had no role in: the design and conduct of the study; collection, management, analysis, and interpretation of the data; preparation, review, or approval of the manuscript; and decision to submit the manuscript for publication. The corresponding author had full access to all the study data and had final responsibility to submit for publication.

## Results

A total of 398 twin pregnancies were included, with 796 live-born infants of whom 214 were MC and 582 were DC. Overall, 3954 ultrasound measurements were included (1877 for MC twins and 2077 for DC twins). There was a median of 10 (interquartile range 8–11) ultrasound scans per fetus in MC twins and 2 (1-6) in DC twins. The maternal and fetal characteristics were displayed in Table 1. The average maternal age was 29.8 ± 2.8 years. About 90% of the women were nulliparous, and 54.0% conceived by in vitro fertilization, of which 11.2% for MC and 69.8% for DC twin pregnancies. The average gestational age at delivery was 36.5 ± 1.2 weeks, and the average birth weight of the infants was 2567 ± 344 g, in which MC twins were found to be delivered earlier and smaller at birth.

**Table 1:** Maternal and fetal characteristics of the twin pregnancies by chorionicity

characteristic	MC twins	DC twins	All
Mother	*n* = 107	*n* = 291	*n* = 398
Maternal age, yrs	28.7 ± 2.9	30.2 ± 2.6	29.8 ± 2.8
Nulliparous	87(81.3)	269(92.4)	356(89.5)
Height, m	1.62 ± 0.04	1.62 ± 0.04	1.62 ± 0.04
Weight, kg	54.3 ± 7.2	56.5 ± 9.0	55.9 ± 8.6
Conception methods			
Conceived naturally	93(86.9)	52(17.9)	145(36.4)
Ovulation induction	2(1.9)	36(12.4)	38(9.6)
*In vitro* fertilization	12(11.2)	203(69.8)	215(54.0)
Ultrasound scans	10(8,11)	2(1,6)	5(1,8)
Fetus	n = 214	n = 582	n = 796
Gestational age at delivery, wks	35.9 ± 1.2	36.7 ± 1.1	36.5 ± 1.2
Birthweight, g	2453.9 ± 361.6	2608.6 ± 328.4	2567.0 ± 344.3
Sex			
Male	104(48.6)	312(53.6)	416(52.3)
Female	110(51.4)	270(46.4)	380(47.7)

**Fig. 3:**
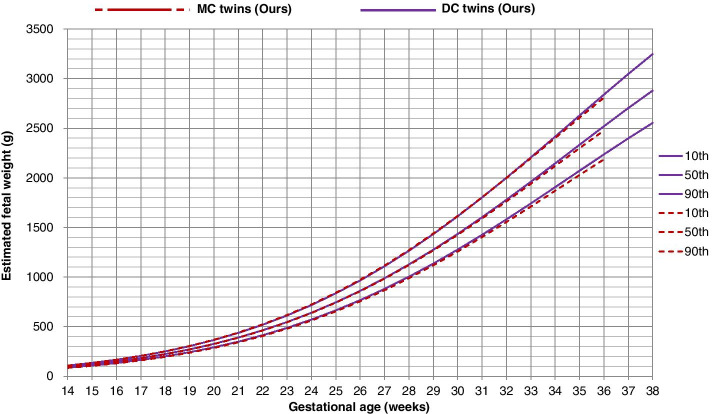
Growth chart for MC and DC twins in the present study (linear mixed model)

As it is shown from the growth chart (Fig. 3), the MC twins were consistently lighter than the DC twins, but the difference was pretty small throughout the whole gestation. Thus, for simplicity, we built only one combined growth standard for both MC and DC twins using a linear mixed model. The weight percentiles for twin fetuses by gestational age were presented in Table 2 and the percentiles for fetal biometric measurements (BPD, HC, AC, and FL) in Table 3. In the sub-population sensitivity analysis, we found that twin fetuses with mothers conceived naturally were a little lighter than those with mothers conceived by in vitro fertilization, similarly, the difference was pretty small throughout the whole gestation (Supplementary Figure S1).

**Table 2:** Weight percentiles for twin fetuses by gestational age

Gestational age, wks	Weight percentiles, g								
	3rd	5th	10th	25th	50th	75th	90th	95th	97th
MC twins									
14	79	81	84	89	95	102	108	112	115
15	99	101	105	111	119	127	135	140	143
16	122	125	130	138	147	157	167	173	177
17	150	154	160	170	181	194	206	213	218
18	184	188	195	207	222	237	252	261	267
19	223	228	237	252	269	287	305	316	324
20	269	275	285	303	324	346	368	381	390
21	322	330	342	363	388	414	440	456	467
22	383	392	406	431	461	493	523	542	555
23	452	462	479	509	544	581	617	640	655
24	530	542	562	596	637	681	723	750	767
25	616	631	654	694	742	793	842	872	893
26	712	729	756	802	857	916	972	1008	1031
27	818	837	867	921	984	1051	1116	1156	1183
28	932	954	988	1049	1121	1198	1271	1318	1348
29	1055	1079	1119	1187	1269	1355	1439	1491	1526
30	1185	1213	1257	1334	1425	1523	1616	1675	1714
31	1323	1354	1403	1489	1591	1699	1804	1869	1913
32	1466	1500	1555	1650	1763	1883	1998	2071	2119
33	1614	1651	1711	1816	1939	2072	2199	2278	2331
34	1763	1804	1870	1984	2119	2264	2402	2489	2547
35	1914	1958	2029	2153	2300	2456	2606	2700	2763
36	2062	2110	2186	2320	2478	2646	2808	2909	2977
DC twins									
14	83	84	87	92	97	103	108	112	114
15	103	105	108	114	121	128	135	139	142
16	127	130	134	141	149	158	167	172	176
17	156	159	164	173	183	195	205	212	216
18	190	194	200	211	224	237	250	258	264
19	230	235	242	256	271	288	303	313	320
20	277	283	292	308	326	346	365	377	385
21	331	338	349	368	390	414	437	451	461
22	392	401	414	437	463	492	519	536	547
23	462	472	488	515	546	580	612	632	646
24	541	553	571	603	640	680	718	741	757
25	629	643	664	701	745	791	835	863	881
26	727	743	767	810	861	914	966	998	1019
27	834	852	880	930	988	1050	1109	1146	1171
28	950	971	1003	1060	1126	1197	1265	1307	1335
29	1075	1099	1136	1200	1276	1356	1433	1481	1513
30	1209	1235	1277	1349	1435	1526	1613	1667	1703
31	1350	1379	1426	1507	1603	1705	1802	1863	1904
32	1497	1529	1581	1672	1779	1892	2001	2069	2114
33	1649	1685	1742	1842	1960	2086	2206	2281	2331
34	1804	1843	1906	2016	2146	2284	2416	2499	2554
35	1960	2003	2072	2192	2333	2484	2628	2718	2778
36	2115	2162	2237	2367	2520	2683	2840	2937	3002
37	2268	2318	2398	2538	2703	2879	3047	3152	3223
38	2414	2468	2554	2704	2880	3068	3248	3361	3436
MC & DC twins									
14	81	83	86	91	96	102	108	112	114
15	101	104	107	113	120	128	135	139	142
16	125	128	132	140	149	158	167	173	176
17	154	158	163	172	183	194	205	212	217
18	188	192	199	210	223	237	251	259	265
19	228	233	241	255	271	288	304	314	321
20	275	281	290	307	326	347	366	379	387
21	328	335	347	367	390	415	438	453	463
22	390	398	412	435	463	492	521	538	550
23	460	470	486	514	546	581	614	635	649
24	538	550	569	602	640	681	720	744	761
25	626	640	662	700	745	792	838	867	886
26	724	739	765	809	861	916	969	1002	1024
27	830	848	877	928	988	1052	1112	1150	1176
28	946	967	1000	1058	1126	1199	1268	1312	1341
29	1071	1094	1132	1198	1275	1358	1436	1486	1519
30	1203	1230	1272	1347	1434	1527	1616	1671	1708
31	1343	1373	1421	1504	1601	1705	1805	1867	1908
32	1489	1523	1575	1667	1776	1891	2002	2071	2118
33	1640	1677	1735	1836	1956	2084	2206	2282	2333
34	1793	1834	1897	2009	2140	2280	2414	2498	2554
35	1948	1992	2061	2182	2325	2478	2624	2715	2776
36	2101	2148	2223	2355	2509	2674	2832	2931	2997
37	2251	2302	2382	2523	2689	2866	3036	3142	3213
38	2395	2449	2535	2685	2862	3051	3232	3345	3421

*Note*: *MC* monochorionic, *DC* dichorionic

**Table 3:** Percentiles for twin fetal sonography measurements by gestational age

Gestational age, wks	Percentiles								
	3rd	5th	10th	25th	50th	75th	90th	95th	97th
Biparietal diameter, mm (MC & DC twins)									
14	25.9	26.2	26.7	27.4	28.3	29.1	30.0	30.5	30.8
15	28.7	29.0	29.5	30.3	31.3	32.2	33.1	33.7	34.0
16	31.5	31.9	32.4	33.3	34.3	35.4	36.4	37.0	37.4
17	34.5	34.8	35.4	36.4	37.5	38.7	39.8	40.4	40.8
18	37.5	37.9	38.5	39.6	40.8	42.0	43.2	43.9	44.4
19	40.5	40.9	41.6	42.7	44.1	45.4	46.7	47.4	47.9
20	43.5	44.0	44.7	46.0	47.4	48.8	50.1	51.0	51.5
21	46.6	47.1	47.8	49.2	50.6	52.2	53.6	54.5	55.1
22	49.6	50.1	50.9	52.3	53.9	55.5	57.1	58.0	58.6
23	52.6	53.1	54.0	55.5	57.1	58.9	60.5	61.4	62.1
24	55.5	56.1	57.0	58.5	60.3	62.1	63.8	64.8	65.5
25	58.3	58.9	59.9	61.5	63.3	65.2	67.0	68.1	68.8
26	61.1	61.7	62.7	64.4	66.3	68.3	70.1	71.2	72.0
27	63.7	64.3	65.4	67.1	69.1	71.2	73.1	74.3	75.0
28	66.2	66.9	68.0	69.8	71.8	74.0	76.0	77.2	78.0
29	68.6	69.3	70.4	72.3	74.4	76.6	78.7	79.9	80.7
30	70.9	71.6	72.7	74.7	76.9	79.1	81.2	82.5	83.4
31	73.0	73.8	74.9	76.9	79.2	81.5	83.7	85.0	85.8
32	75.0	75.8	77.0	79.0	81.3	83.7	85.9	87.3	88.2
33	76.9	77.7	78.9	81.0	83.4	85.8	88.1	89.4	90.3
34	78.7	79.5	80.7	82.9	85.3	87.7	90.0	91.4	92.4
35	80.4	81.2	82.4	84.6	87.0	89.6	91.9	93.3	94.3
36	81.9	82.7	84.0	86.2	88.7	91.3	93.6	95.1	96.1
37	83.4	84.2	85.5	87.7	90.3	92.9	95.3	96.8	97.7
38	84.8	85.6	87.0	89.2	91.8	94.4	96.8	98.3	99.3
Head circumference, mm (MC & DC twins)									
14	92.4	93.2	94.6	96.8	99.4	102.1	104.6	106.1	107.1
15	102.6	103.5	105.0	107.5	110.4	113.4	116.1	117.7	118.8
16	113.2	114.2	115.8	118.6	121.8	125.0	128.0	129.8	131.0
17	124.1	125.2	127.0	130.0	133.4	137.0	140.2	142.2	143.5
18	135.2	136.4	138.3	141.6	145.3	149.2	152.7	154.8	156.3
19	146.4	147.7	149.8	153.3	157.3	161.5	165.3	167.6	169.1
20	157.7	159.1	161.3	165.1	169.4	173.8	177.9	180.4	182.0
21	168.9	170.4	172.8	176.8	181.4	186.1	190.5	193.1	194.8
22	180.0	181.6	184.1	188.4	193.3	198.2	202.9	205.7	207.5
23	190.9	192.6	195.2	199.8	204.9	210.1	215.0	218.0	219.9
24	201.5	203.3	206.1	210.8	216.2	221.7	226.8	230.0	232.0
25	211.8	213.7	216.6	221.6	227.2	233.0	238.3	241.5	243.7
26	221.7	223.7	226.7	231.9	237.7	243.8	249.3	252.7	254.9
27	231.2	233.3	236.4	241.8	247.8	254.1	259.8	263.3	265.6
28	240.3	242.4	245.6	251.2	257.4	263.9	269.8	273.5	275.8
29	248.8	251.0	254.3	260.1	266.5	273.2	279.3	283.0	285.5
30	256.9	259.1	262.6	268.4	275.1	281.9	288.2	292.0	294.5
31	264.6	266.8	270.3	276.3	283.2	290.1	296.6	300.5	303.1
32	271.7	274.0	277.6	283.7	290.7	297.8	304.4	308.4	311.0
33	278.4	280.8	284.4	290.7	297.8	305.0	311.7	315.8	318.5
34	284.7	287.1	290.8	297.2	304.4	311.8	318.6	322.8	325.5
35	290.7	293.1	296.9	303.3	310.7	318.1	325.1	329.3	332.0
36	296.3	298.7	302.6	309.1	316.5	324.1	331.1	335.4	338.2
37	301.6	304.1	308.0	314.6	322.1	329.8	336.9	341.2	344.1
38	306.8	309.3	313.2	319.9	327.5	335.3	342.5	346.8	349.7
Abdominal circumference, mm (MC & DC twins)									
14	77.8	78.7	80.1	82.5	85.3	88.2	90.9	92.5	93.6
15	86.8	87.8	89.4	92.1	95.2	98.4	101.4	103.2	104.4
16	96.3	97.4	99.1	102.1	105.5	109.1	112.4	114.4	115.7
17	106.0	107.2	109.1	112.4	116.2	120.0	123.6	125.9	127.3
18	116.0	117.3	119.4	123.0	127.1	131.3	135.2	137.6	139.2
19	126.1	127.6	129.8	133.7	138.1	142.7	146.9	149.5	151.2
20	136.4	138.0	140.4	144.5	149.3	154.2	158.8	161.6	163.4
21	146.7	148.4	151.0	155.4	160.5	165.7	170.6	173.6	175.6
22	157.0	158.7	161.5	166.2	171.7	177.2	182.4	185.6	187.7
23	167.1	169.0	172.0	177.0	182.7	188.6	194.1	197.5	199.7
24	177.2	179.2	182.3	187.6	193.6	199.9	205.7	209.2	211.6
25	187.1	189.1	192.4	198.0	204.3	210.9	217.0	220.7	223.2
26	196.7	198.9	202.3	208.1	214.8	221.7	228.0	231.9	234.5
27	206.2	208.5	212.0	218.1	225.0	232.2	238.8	242.9	245.5
28	215.4	217.8	221.4	227.8	235.0	242.4	249.3	253.5	256.3
29	224.4	226.8	230.7	237.2	244.7	252.4	259.5	263.9	266.8
30	233.2	235.7	239.6	246.4	254.1	262.1	269.5	274.0	277.0
31	241.7	244.3	248.4	255.4	263.4	271.6	279.2	283.8	286.9
32	250.1	252.8	257.0	264.2	272.4	280.9	288.7	293.5	296.7
33	258.5	261.2	265.5	272.9	281.3	290.0	298.1	303.0	306.3
34	266.7	269.5	274.0	281.6	290.2	299.1	307.4	312.4	315.8
35	275.0	277.9	282.4	290.2	299.1	308.2	316.7	321.9	325.3
36	283.3	286.3	291.0	298.9	308.0	317.4	326.1	331.4	334.9
37	291.8	294.9	299.7	307.8	317.1	326.7	335.6	341.1	344.7
38	300.6	303.8	308.7	317.0	326.6	336.4	345.5	351.1	354.7
Femur length, mm (MC & DC twins)									
14	13.1	13.3	13.6	14.1	14.7	15.3	15.9	16.3	16.5
15	15.4	15.6	15.9	16.5	17.2	17.9	18.6	19.0	19.2
16	17.8	18.0	18.4	19.1	19.8	20.6	21.4	21.8	22.1
17	20.3	20.5	21.0	21.7	22.6	23.5	24.3	24.8	25.2
18	22.9	23.2	23.6	24.5	25.4	26.4	27.4	27.9	28.3
19	25.5	25.9	26.4	27.3	28.3	29.4	30.5	31.1	31.5
20	28.2	28.6	29.1	30.1	31.3	32.5	33.6	34.2	34.7
21	30.9	31.3	31.9	32.9	34.2	35.4	36.6	37.4	37.8
22	33.5	33.9	34.6	35.7	37.0	38.4	39.7	40.4	40.9
23	36.1	36.5	37.2	38.4	39.8	41.2	42.6	43.4	43.9
24	38.5	39.0	39.7	41.0	42.5	44.0	45.4	46.2	46.8
25	40.9	41.4	42.2	43.5	45.0	46.6	48.0	48.9	49.5
26	43.2	43.7	44.5	45.8	47.4	49.0	50.5	51.5	52.1
27	45.3	45.8	46.6	48.1	49.7	51.4	52.9	53.9	54.5
28	47.3	47.9	48.7	50.2	51.8	53.5	55.1	56.1	56.7
29	49.2	49.8	50.7	52.1	53.8	55.6	57.2	58.2	58.8
30	51.1	51.6	52.5	54.0	55.7	57.5	59.2	60.2	60.8
31	52.8	53.4	54.3	55.8	57.5	59.3	61.0	62.0	62.7
32	54.5	55.1	56.0	57.5	59.3	61.1	62.8	63.8	64.5
33	56.2	56.8	57.7	59.3	61.0	62.9	64.6	65.6	66.3
34	57.9	58.5	59.4	61.0	62.8	64.6	66.3	67.4	68.1
35	59.6	60.2	61.2	62.8	64.6	66.4	68.2	69.2	69.9
36	61.5	62.1	63.0	64.6	66.5	68.3	70.1	71.1	71.8
37	63.5	64.1	65.0	66.6	68.5	70.4	72.1	73.2	73.9
38	65.6	66.3	67.2	68.9	70.7	72.7	74.4	75.5	76.2

*Note*: *MC* monochorionic, *DC* dichorionic

**Fig. 4:**
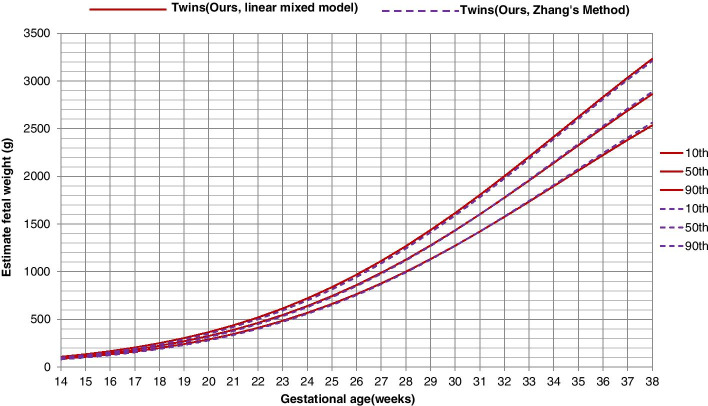
Growth chart for twins built from linear mixed model and that built from Zhang’s method

**Fig. 5:**
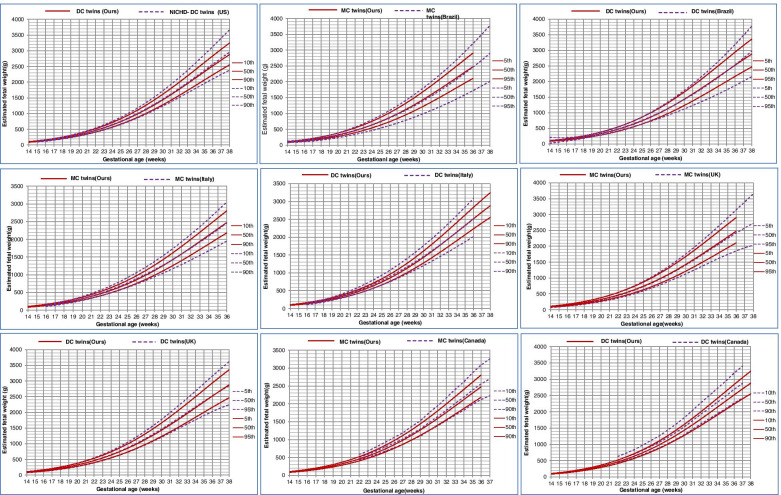
Comparison of growth chart for our twins and that of Fetal Growth Studies from the NICHD (Grantz KL, 2016), Brazil (Araujo Júnior E, 2014), Italy (Ghi T, 2017), UK (Stirrup OT, 2016), and Canada (Shivkumar S, 2015)

When comparing the chart with that built using Zhang’s method (weight percentiles displayed in Supplementary Table S2), we found that the two charts almost overlapped except that the Zhang’s curve was slightly lower in the 90th percentiles (Fig. 4). Furthermore, we compared our charts with those from previous studies based on different populations. Compared to those from the US (NICHD study) [8], Brazil [15], Italy [16] and UK [17], the Chinese twins had very similar 50th percentiles, but higher 5th and 10th percentiles and lower 90th and 95th percentiles, especially in late gestation (> 28 weeks or > 32 weeks; Fig. 5). The only exception is that the 10th percentiles for Chinese MC and DC twins almost overlapped with those from Canada, however, the Chinese twins had lower 50th and much lower 90th percentiles, especially in late gestation (Fig. 5) [14].

Compared with the Hadlock singleton standard, the application of our growth chart to live births of the source cohort resulted in a much lower proportion of SGA (< 10th) (26.9% for Hadlock’s vs 16.1% for our growth chart). When applying our growth chart, the ORs of neonatal death and adverse perinatal outcomes for SGA compared with non-SGA [3.49 (95%CI: 0.58, 20.99) and 3.74 (95%CI: 2.85, 4.92), respectively] were substantially elevated relative to the Hadlock’s standard [1.81(0.30, 10.91) and 2.30(1.79, 2.94), respectively]. And the ORs increased slightly when the analyses were restricted to those without birth defects (Table 4).

**Table 4:** Comparison of the ability of the growth chart in predicting adverse perinatal outcomes

Grow chart	Perinatal outcomes	Among SGA	Among non-SGA	OR (95% CI)	AUC (95% CI)
		n (%)	n (%)		
MC & DC twins	All live births	*n* = 309	*n* = 1611		
	Neonatal death	2(0.7)	3(0.2)	3.49(0.58, 20.99)	0.620(0.380, 0.860)
	Adverse events ^a^	113(36.6)	215(13.4)	3.74(2.85, 4.92)	0.611(0.584, 0.638)
	Birth without defect	*n* = 267	*n* = 1525		
	Neonatal death	2(0.75)	3(0.2)	3.82(0.64, 22.95)	0.626(0.386, 0.866)
	Adverse events ^a^	84(31.5)	146(9.6)	4.32(3.17, 5.89)	0.624(0.592, 0.656)
Hadlock	All live births	*n* = 516	*n* = 1404		
	Neonatal death	2(0.4)	3(0.2)	1.81(0.30,10.91)	0.566(0.326. 0.806)
	Adverse events ^a^	137(26.6)	191(13.6)	2.30(1.79, 2.94)	0.590(0.561, 0.619)
	Birth without defect	*n* = 469	*n* = 1323		
	Neonatal death	2(0.4)	3(0.23)	1.88(0.31,11.27)	0.569(0.329, 0.809)
	Adverse events ^a^	106(22.6)	124(9.4)	2.81(2.12, 3.74)	0.614(0.580, 0.648)

^a^Adverse events including: Neonatal death / Neonatal intensive care unit (NICU) ≥ 14d / Transfer to higher-level or special care unit

## Discussion

### Principle findings

In this prospective study, we constructed a normal fetal growth standard for Chinese twins. The MC twins were consistently lighter than the DC twins but the differences were very small throughout the gestation. The growth chart built using linear mixed model was comparable to that by Zhang’s method [18]. Overall, Chinese twins had almost identical the 50th percentiles to those reported in previous studies, but tended to have a narrower range between the 10th and 90th (5th and 95th) percentiles in late gestation (> 28 weeks or > 32 weeks).

### Comparison with previous studies in twin pregnancies

The construction of a fetal growth chart relies much on the population that the study selects and the statistical method that it adopts. To obtain an optimal fetal growth standard, we selected only healthy twin pregnancies, which was similar to most previous studies [8, 14, 15] except one use unselected pregnancies [17], and another one further excluded twin with a birthweight below the 5th percentile of their national singleton standard [16]. To construct a standard, we used a stricter inclusion criteria than other studies [14–16], i.e. pregnancies with unmatched Us-GA and LMP-GA, or maternal age < 20 years or > 35 years, or crown-rump length discordance > 10%, or sIUGR were all excluded, which was different from most previous studies [14–16]. When modeling fetal growth for twins, the dependency of data, namely, the clustering of the twins and serial measurements on the same twin, should be taken into consideration. The present study used linear mixed model accounting for data correlation from both mother-level and fetus-level, which was also considered in most previous studies [8, 14, 16, 17], but was not in the Brazil study that used polynomial regression [15].

When comparing our charts with those from previous studies, we found that the Chinese twins had very similar 50th percentiles, but higher 5th and 10th percentiles and lower 90th and 95th percentiles especially in late gestation [8, 14–17]. The difference may originate from several aspects. Firstly, it is now well recognized that the difference in fetal growth is largely due to biological differences among regions and ethnicities [26]. Some of the previous studies were multicenter or included several ethnicities, which would lead to larger range. However, the present study included only Chinese twin pregnancies, and most of them were Han ethnicity, and few of them were too thin or too heavy. Thus, the population may be genetically and physically more homogenous, which would make the growth percentiles narrower. Secondly, the study design may also play an important role. Some of the previous studies used unselected twin pregnancies [17], who might have had more complications (i.e. sIUGR or TTTS) and larger variation in fetal growth, resulting in a wider range for fetal growth reference. As the present study aimed to construct an optimal growth standard, healthy twin-pregnancies with stricter definition were selected, who were likely to have smaller variation and a narrower range. Also, since the fetal growth standard is gestational-age-dependent, the exclusion of women with inaccurate GA (unmatched Us-GA and LMP-GA) can lead to a narrower range. Furthermore, repeated measurements on an individual fetus were more homogeneous than those from a cross-sectional study that used only one measurement from the fetus[Brazil]. Finally, our study was conducted in one center and the ultrasound scans were performed by three experienced, well-trained sonographers, whereas some of the previous studies used data from several centers, which would have larger inter-observer variation and wider range for fetal growth. Supplementary Figure S2 indicates low inter-observer variation and good homogeneity.

### Clinical implications

The use of a singleton fetal growth chart to evaluate the twin pregnancies is a very common practice. However, it has been well demonstrated that compared to singletons, the growth of twin fetuses become slower and the fetal growth curves diverge significantly in late gestation (i.e. after 28–32 weeks) [6–8]. Therefore, twins need a separate standards to evaluate their growth and identify growth restriction and adverse prenatal outcomes more accurately. Indeed, when applying the present chart instead of the Hadlock singleton standard to live-born twins, the proportion of SGA identified was more precise, and the risk of adverse events in SGA identified was substantially elevated. When the identification of SGA was more precise, unnecessary medical costs and burden could be avoided. Moreover, given that the Zhang’s curve is very similar to the ones of this study, indicating that Zhang’s method is applicable to Chinese twins, other areas may use Zhang’s method to generate their own curves if deemed necessary. However, prior to a new standard being applied in clinical use, prospective studies are warranted to ensure its performance to identify pregnancies that are at higher risk of adverse perinatal outcomes.

### Strength and limitation

The present study has several strengths. Firstly, all the materials were from a prospectively-designed cohort study, which enabled us to obtain the information with minimal bias. Secondly, gestational age was ascertained by first-trimester CRL of the larger twin, and those with unmatched Us-GA and LMP-GA were excluded from the present study. By doing so, the accuracy of gestational age was ensured. Thirdly, all the ultrasound scans were conducted by three experienced sonographers, and ultrasound biometry was measured according to the same standard operating procedure. Fourth, the linear mixed model, which took the correlation within the twin-pair and serial measurements of a single fetus into account, provided a better estimation of the fetal growth for twin pregnancies.

Still, there are several limitations that we should acknowledge. First, the study was conducted in a single tertiary center, which might limit the generalizability of its results. However, most twin pregnancies are commonly referred and delivered at tertiary hospitals in China. As a tertiary hospital of about 30,000 deliveries per year in Shanghai, our study population should be of good representativeness. Indeed, though 69% of our study subjects were from east of China, our study subjects covered 88% (30/34) of provinces in China. Thus, our results can be applied at least to twin pregnancies in the east part of China. Other areas may generate their own curves by Zhang’s method given that the Zhang’s curve is very similar to the ones of this study. Finally, although the ability of the growth chart in identifying small fetuses at risk of neonatal death and adverse perinatal outcomes appeared to be good, future studies with long-term follow up are needed to determine the best cut-point in predicting long-term fetal outcomes-the ultimate goal of monitoring fetal growth.

## Conclusion

We created a fetal growth chart for Chinese twins. The MC twins were consistently lighter than the DC twins but with small differences throughout the gestation. Overall, the Chinese twins were identical to previous studies in the 50th percentiles, but tended to have narrower ranges at late gestation. Our standard performed much better than the Hadlock’s standard in predicting low birth weight infants associated with adverse perinatal outcomes in twin pregnancies. Our study also indicates that Zhang’s method is applicable to Chinese twins in generating fetal growth reference.

## Supplementary Information

**Additional file 1:** **Supplementary Table S1**. Akaike information criteria (AIC) value and residual standard errors for the models underwent selection. **Supplementary Table S2**. Weight percentiles for twin fetuses by gestational age built from Zhang’s methods. **Supplementary Figure S1**. Growth chart for twins conceived naturally and twins conceived by in vitro fertilization. **Supplementary Figure S2**. Mean estimate fetal weight by gestational age for three sonographers

## Data Availability

The datasets used and/or analyzed during the current study are available from the corresponding author on reasonable request.

## Abbreviations

EFW: Estimated fetal weight
BPD: Biparietal diameter
HC: Head circumference
AC: Abdominal circumference
FL: Femur length
MC: Monochorionic
MCDA: Monochorionic diamniotic
DC: Dichorionic
DCDA: Dichorionic diamniotic
SGA: Small for gestational age
LMP-GA: Last menstrual period-based gestational age
Us-GA: Ultrasound-estimated gestational age
