# Supplementary material for: Fetal growth standards for Chinese twin pregnancies
Source: BMC Pregnancy Childbirth. 2021 Jun 22;21:436. doi: 10.1186/s12884-021-03926-y (PMC8220745; doi:10.1186/s12884-021-03926-y)
Supplement: Supplementary file 1 — Additional file 1: Supplementary Table S1. Akaike information criteria (AIC) value and residual standard errors for the models underwent selection. Supplementary Table S2. Weight percentiles for twin fetuses by gestational age built from Zhang’s methods. Supplementary Figure S1. Growth chart for twins conceived naturally and twins conceived by in vitro fertilization. Supplementary Figure S2. Mean estimate fetal weight by gestational age for three sonographers [file 12884_2021_3926_MOESM1_ESM.zip › Supplementary Figure S2.pptx]

## Slide 1
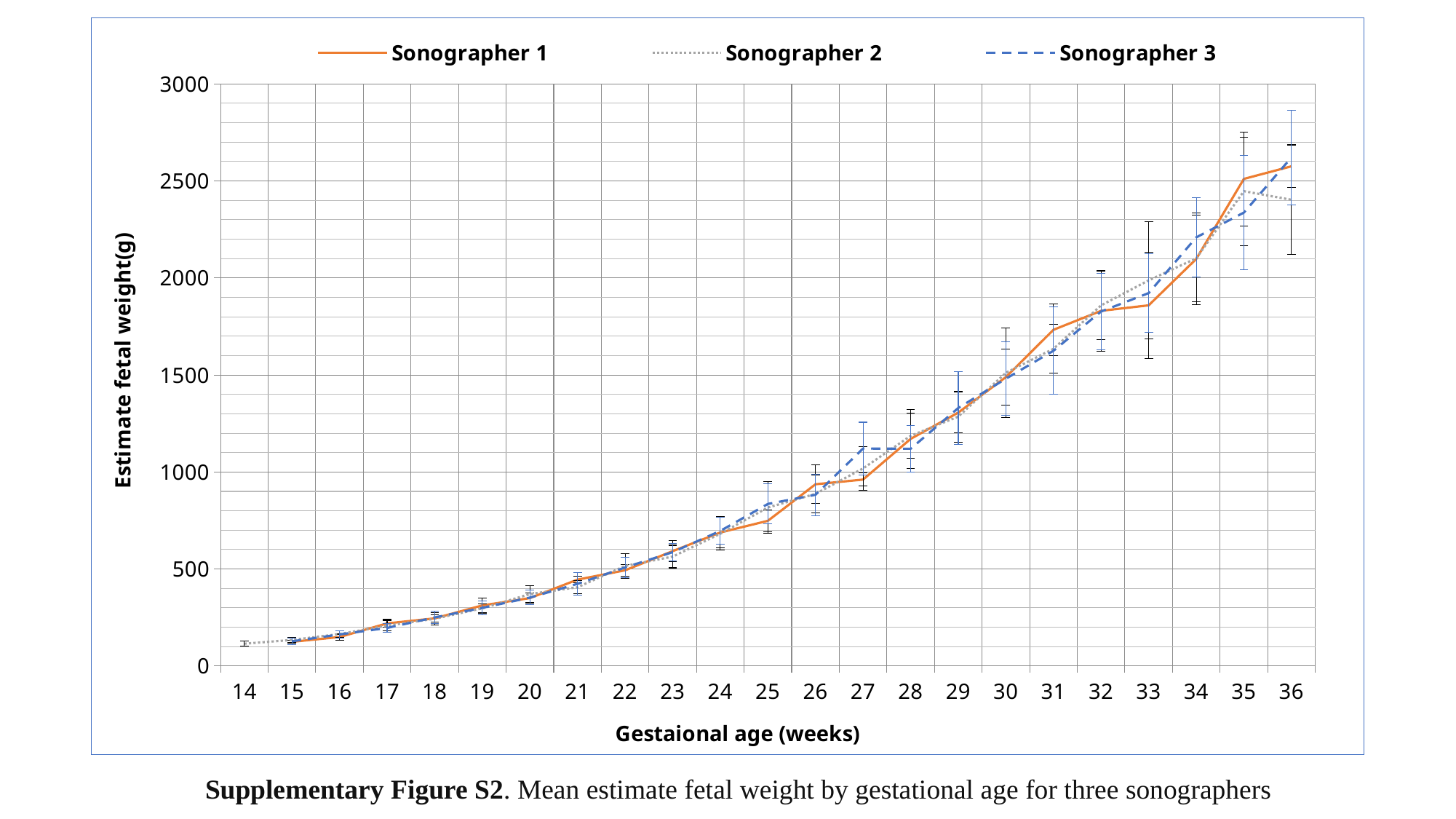

### Chart
| Category | Sonographer 1 | Sonographer 2 | Sonographer 3 |
|---|---|---|---|
| 14 | None | 114.8 | None |
| 15 | 124.6 | 134.3 | 127.1 |
| 16 | 149.4 | 164.8 | 162.7 |
| 17 | 219.7 | 208.2 | 196.2 |
| 18 | 245.5 | 243.6 | 250.4 |
| 19 | 312.5 | 297.5 | 299.6 |
| 20 | 350.4 | 370.8 | 352.9 |
| 21 | 445.5 | 406.2 | 424.0 |
| 22 | 493.5 | 515.8 | 507.8 |
| 23 | 592.3 | 564.2 | 586.9 |
| 24 | 688.3 | 682.0 | 696.4 |
| 25 | 748.5 | 816.9 | 835.2 |
| 26 | 936.7 | 887.4 | 882.5 |
| 27 | 960.9 | 1018.3 | 1120.8 |
| 28 | 1170.7 | 1186.5 | 1119.8 |
| 29 | 1306.1 | 1285.4 | 1329.8 |
| 30 | 1488.9 | 1511.0 | 1480.7 |
| 31 | 1732.7 | 1634.7 | 1624.6 |
| 32 | 1830.6 | 1858.8 | 1827.2 |
| 33 | 1858.9 | 1987.8 | 1922.2 |
| 34 | 2098.1 | 2101.3 | 2209.3 |
| 35 | 2510.6 | 2447.2 | 2336.8 |
| 36 | 2575.4 | 2402.9 | 2621.3 |Supplementary Figure S2. Mean estimate fetal weight by gestational age for three sonographers
